# Defined Small Molecules Produced by Himalayan Medicinal Plants Display Immunomodulatory Properties

**DOI:** 10.3390/ijms19113490

**Published:** 2018-11-06

**Authors:** Phurpa Wangchuk, Simon H. Apte, Michael J. Smout, Penny L. Groves, Alex Loukas, Denise L. Doolan

**Affiliations:** 1 Centre for Molecular Therapeutics, Australian Institute of Tropical Health and Medicine, James Cook University, Cairns, QLD 4878, Australia; michael.smout@jcu.edu.au (M.J.S.); alex.loukas@jcu.edu.au (A.L.); denise.doolan@jcu.edu.au (D.L.D.); 2 QIMR Berghofer Medical Research Institute, Brisbane, QLD 4006, Australia; Simon.Apte@health.qld.gov.au (S.H.A.); Penny.Groves@qimrberghofer.edu.au (P.L.G.)

**Keywords:** medicinal plants, phytochemicals, scoulerine, bergapten, immunomodulator, adjuvant, cytoxicity, dendritic cells, immune modulation

## Abstract

Plant-derived compounds that modulate the immune responses are emerging as frontline treatment agents for cancer, infectious diseases and autoimmunity. Herein we have isolated 40 phytochemicals from five Bhutanese *Sowa Rigpa* medicinal plants—*Aconitum laciniatum*, *Ajania nubegina*, *Corydalis crispa*, *Corydalis dubia* and *Pleurospermum amabile*—and tested 14 purified compounds for their immunomodulatory properties using a murine dendritic cell (DC) line, and cytotoxicity against a human cholangiocyte cell line using xCELLigence real time cell monitoring. These compounds were: pseudaconitine, 14-veratryolpseudaconitine, 14-*O*-acetylneoline, linalool oxide acetate, (*E*)-spiroether, luteolin, luteolin-7-*O*-β-d-glucopyranoside, protopine, ochrobirine, scoulerine, capnoidine, isomyristicin, bergapten, and isoimperatorin. Of the 14 compounds tested here, scoulerine had adjuvant-like properties and strongly upregulated *MHC-I* gene and protein expression whereas bergapten displayed immunosuppressive properties and strongly down-regulated gene and protein expression of MHC-I and other co-stimulatory molecules. Both scoulerine and bergapten showed low cytotoxicity against normal healthy cells that were consistent with their immunoregulatory properties. These findings highlight the breadth of immunomodulatory properties of defined compounds from Bhutanese medicinal plants and show that some of these compounds exert their mechanisms of action by modulating DC activity.

## 1. Introduction

The role of plants in preventing and healing ailments has been known since antiquity. Plant-derived natural products are used in both modern and traditional medicines for treating various diseases including but not limited to cancer, malaria, cardiovascular and Alzheimer’s diseases [1,2,3]. The earliest records show that Mesopotamians (2600 B.C.) used *Cupressus* and *Commiphora* species for treating coughs, colds and inflammation, and the plants are still used to this day for treating the same conditions. Ancient Egyptians (*Ebers Papyrus*), Chinese (*Huangdi Neijing*), Indians (*Ayurvedic*), Greeks (*Hippocratic-Galenic*), Romans (*Greco-Roman*), Arabs (*Unani-Tibb*), and Bhutanese (*Sowa Rigpa*) used plants in crude forms as traditional medicines, home remedies, potions and oils [2]. These crude drugs or crude extracts from plants contain many complex compounds with therapeutic properties. For example, the crude bark extract from cinchona tree was used for treating malaria since 1632 and later in 1820, a pure antimalarial compound (quinine) was isolated, which marked the first successful use of a chemical compound in modern medicine to treat infectious diseases [4]. In 1897, aspirin was first manufactured as a synthetic analogue of salicylic acid that was isolated from a willow tree [5], paving the way to the now multi-billion dollar synthetic pharmaceutical industry.

Recent advances in natural product chemistry, spectroscopic and bioscreening technologies have reinvigorated the use of natural products for human health. Today, both naturally-derived and chemically synthesized drugs are used with a tremendous impact on disease prevention and treatment, resulting in the saving of countless lives. Overall, of the 1184 drugs/leads or new chemical entities approved by the U.S Food and Drug Administration between 1981 and 2006, 52% were natural products and their derivatives, and only 30% were of synthetic origin [6]. Moreover, about 11% of the 252 essential drugs currently listed by the World Health Organization (WHO) are exclusively of plant origin, and 80% of those plant-derived drugs were discovered from medicinal plants used in traditional herbal medicines [7]. Some drugs were discovered using biorational strategy, which exploits the knowledge that plants produce various secondary metabolites in response to injury or attack (e.g., by pathogenic bacteria or fungi). With innovation in bioassay screening tools and life science robotic technologies, it is estimated that the global market for plant-derived drugs will grow from $29.4 billion in 2017 to around $39.6 billion by 2022 [8].

Currently, natural products with immune modulating activities are emerging as frontline treatment agents for cancer, infectious diseases and autoimmunity [9]. Of particular interest in immunomodulation is the effect of compounds on dendritic cells (DCs) activity. DCs provide a link between the innate and the adaptive immune responses and are key orchestrators of the adaptive immune response [9,10]. DCs can be subdivided into interferon-producing plasmacytoid (pDC), monocyte-derived (MoDC), and classical/conventional dendritic cell (cDC), which in mice have been further classified into two major subsets as cDC 1 and cDC2 [11]. While cDC1 is specialized in cross-presentation of CD8^+^ T cells that are critical for immunity against intracellular pathogens, viruses and cancer [12], cDC2 promotes CD4^+^ T cell differentiation into subsets specializing in antiviral, antifungal or helminth immunity [13]. cDC subset composition in humans depends on the lymph node tissue site with lung-draining lymph nodes (LN) having the highest proportion of mature DC compared to other LN sites, and cDC2 subset exhibits predominant maturation phenotypes within LNs compared to cDC1 [14]. Human CD141^+^ and CD1c^+^ cDCs (originally identified in blood) have been ontogenetically aligned to mouse cDC1 and cDC2, respectively [15]. These cDCs have the capacity to acquire antigens in peripheral tissues, deliver them to draining LNs, and undergo maturation through upregulation of major histocompatibility (MHC) and co-stimulatory molecules which are required for T cell activation [14,16].

To become properly activated, T cells require two signals including antigen presentation and costimulatory signals. Antigen presentation requires MHC-I and MHC-II expression on the DC for CD8^+^ T cell and CD4^+^ T cell recognition, respectively. The costimulatory signals are provided by the DC to the T cells such as those mediated by CD80, CD86 and CD28. Compounds that can stimulate DCs to upregulate these molecules have therapeutic potential as they can strongly enhance T cell responses and may make good vaccine adjuvants or immunostimulators. Conversely, compounds that can downregulate DC function may be useful to induce immune tolerance and have therapeutic value for treating autoimmune diseases, allergies and to promote transplant tolerance. Several studies have shown that plant products or extracts can affect the behaviour of DCs [17]. For example, pine cone and *Echinacea pupurea* extracts have been demonstrated to modulate the expression of MHC and stimulatory and transmembrane molecules including MHC-II, CD86, and CD54 on DCs [18,19,20]. Purified secondary metabolites, such as resveratrol isolated from grapes and triptolide isolated from the Chinese medicinal plant *Tripterygium wilfordii* Hook F, have demonstrated suppression of DC maturation and inhibition of the expression of CD80 and CD86 [21,22]. Plant extracts and their secondary metabolites have also been shown to exhibit robust immunomodulatory effects on chronic neuro-inflammation and cognitive aging [23].

We have previously developed a novel high throughput DC-based assay to assess the ability of lipopeptide-based nano-vaccine constructs to modulate DC function [24]. This DC assay utilizes the DC2.4 cell line derived from mouse that is known to present exogenous antigen in the context of both MHC class I and MHC class II molecules and can cross-present antigen to CD8^+^ T cells [25,26,27]. Herein we have applied this DC-based screening assay together with a cell-based cytotoxicity assay [28] to investigate the immunomodulatory and cytotoxic properties of plant compounds isolated from five Bhutanese medicinal plants: *Aconitum laciniatum*, *Ajania nubigena*, *Corydalis crispa*, *C. dubia* and *Pleurospermum amabile*. These five medicinal plants grow in an extreme climatic condition of the Himalayan alpine mountains of Bhutan at an altitude between 3900–4700 m above sea level and they are used in the scholarly Bhutanese *Sowa Rigpa* medicine (BSM) for treating various human health disorders (see Table 1 for individual plant uses) [29]. The BSM has been adapted from Tibetan medicine, which is currently practiced worldwide including Nepal, India, China, Mongolia, Russia, Europe and North America [30]. The plants flower between June–September and that is when the farmers collect them from their natural habitat. The plants are either sun-dried or shade-dried before preparing them into multi-ingredient dosage formulations as powders, capsules, tablets, pills, syrup and ointments.

## 2. Results

### 2.1. Medicinal Plants, Isolated Phytochemicals and Selected Compounds for In Vitro Assay

**Figure 1:**
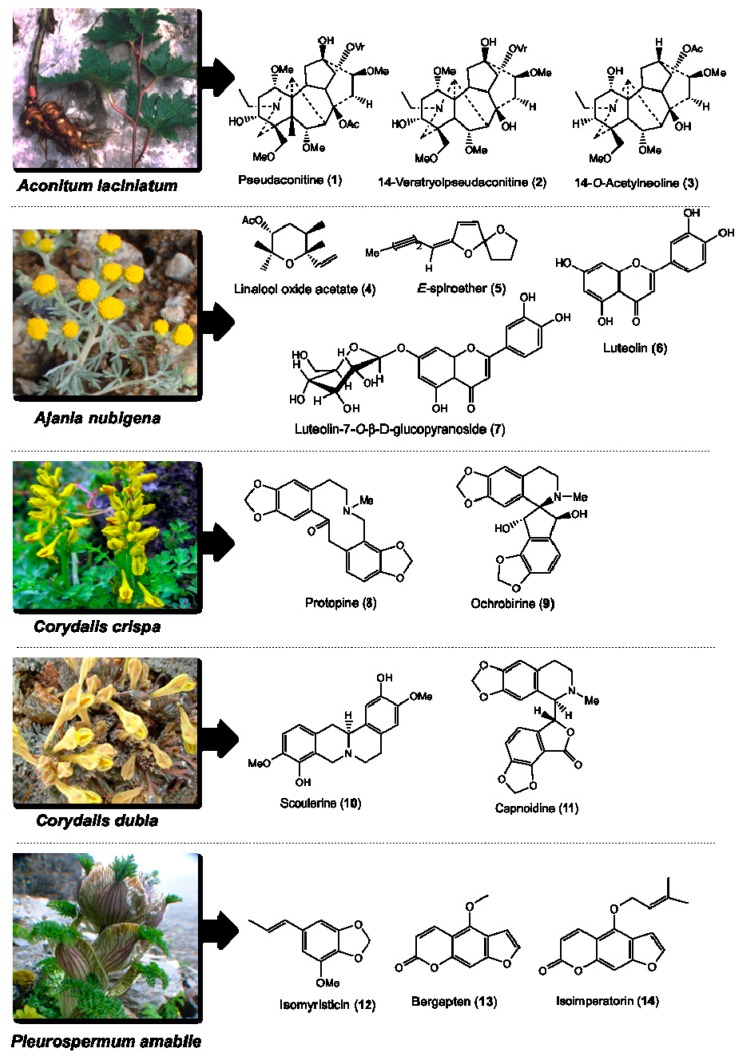
Structures of the compounds isolated from five Bhutanese medicinal plants using methods described by us previously [31,32,33,34,35], which were tested for their immunomodulatory and cytotoxic activities. Representative plant photos of *Aconitum laciniatum*, *Ajania nubigena*, *Corydalis crispa*, *C. dubia*, and *Pleurospermum amabile* are shown.

Five medicinal plants: *A. laciniatum*, *A.* *nubigena*, *C. crispa*, *C. dubia*, and *P. amabile* were collected for their roots, aerial parts and whole plant materials from the high altitude Himalayan alpine mountains of Bhutan (3500–4900 m above sea level). These plants were air-dried, sliced, and extracted using methanol/ethanol, and then subjected to repeated column and preparative thin layer chromatography separations. In total, we isolated 40 compounds belonging to alkaloids, flavonoids, terpenoids, phenylpropanoids and furanocoumarins [31,32,33,34,35]. The plant parts used in the Bhutanese medicine, the parts collected for this study, total number of phytochemicals isolated from each plant, the major classes of phytochemicals of isolated compounds, and the phytochemicals selected for the immunoregulatory and cytotoxicity studies are provided in Table 1. Of the 40 isolated compounds, 14 of them including pseudaconitine (**1**), 14-veratryolpseudaconitine (**2**), 14-*O*-acetylneoline (**3**), linalool oxide acetate (**4**), (*E*)-spiroether (**5**), luteolin (**6**), luteolin-7-*O*-β-d-glucopyranoside (**7**), protopine (**8**), ochrobirine (**9**), scoulerine (**10**), capnoidine (**11**), isomyristicin (**12**), bergapten (**13**), and isoimperatorin (**14**) (Figure 1) were obtained in quantities sufficient for bioactivity screening. Compounds **1**–**3** belong to the phytochemical class of C_19_-diterpenoid alkaloids. Compounds **4**–**7** belong to the phytochemical classes of terpenoids and flavonoids**.** Compounds **8**–**11** belong to benzylisoquinoline type of alkaloids. Compounds **12**–**14** belong to the phytochemical classes of phenylpropanoids and furanocoumarins. Their purity was determined by GC-MS and NMR (data on each compound is given in Section 4).

### 2.2. Plant Compounds Showed Immunomodulatory Activities in Dendritic Cell (DC)-Based Immunoassay

**Figure 2:**
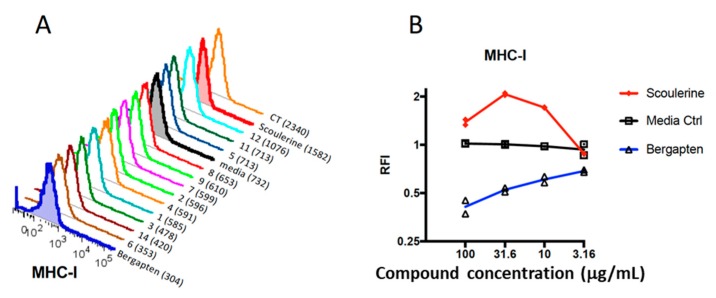
Modulation of immune molecule expression by plant compounds. (**A**) FACS plots showing the median fluorescence intensity (MFI) of MHC-I expression for all selected plant compounds, cells grown in media only, and a control grown with cholera toxin (CT). (**B**) The concentration at which each compound (scoulerine and bergapten) was most active was determined by titrating each compound (four mid-log dilutions starting at 100 µg/mL) and observing the maximal increase (or decrease) in expression of each fluorochrome (Table 2). Data for duplicate assays is presented. Media Ctrl = Media control.

To gain some insight into the immunomodulatory properties of the 14 selected plant compounds, we utilized a dendritic cell (DC)-based immunoassay [24]. Briefly, the 14 selected compounds were prepared in 2% DMSO in cell culture media with 5% FCS (final concentration of 1 mg/mL) and further titrated in cell culture media (6 mid-log dilutions starting) prior to incubation with DC2.4 cells overnight in 96-well plates. The following day, the cells were harvested and prepared for flow cytometric analysis using a panel of defined surface markers including MHC-I, MHC-II, CD40, CD44, CD80, CD86, and CD274 (PDL-1). Dead cells were excluded based on their uptake of a dead cell stain and the potent DC activator cholera toxin (CT) was included as a positive control [37]. In Figure 2A, representative FACS plots for MHC-I expression are shown for all compounds. Notably, the Median Fluorescence Intensity (MFI) showed that compound 13 (bergapten, MFI 304) induced down-regulation of surface MHC-I expression compared to the media only control (MFI 732); this contrasts with compound 10 (scoulerine), which induced an increase in MHC-I expression (MFI 1582). For all compounds and surface markers tested, we calculated the relative fluorescence intensity (RFI) by dividing the MFI of the test sample with the appropriate MFI of the media only control. We next determined the optimal concentration of the compounds by observing the maximal increase (or decrease) in protein expression; for example, for MHC-I, scoulerine (**10**) was maximal at the concentration of 31.6 µg/mL, whereas bergapten (**13**) was most active (with respect to downregulation of MHC-I) at the 100 µg/mL (Figure 2B, Table 2).

### 2.3. Modulation of Gene Expression by Plant Compounds

**Figure 3:**
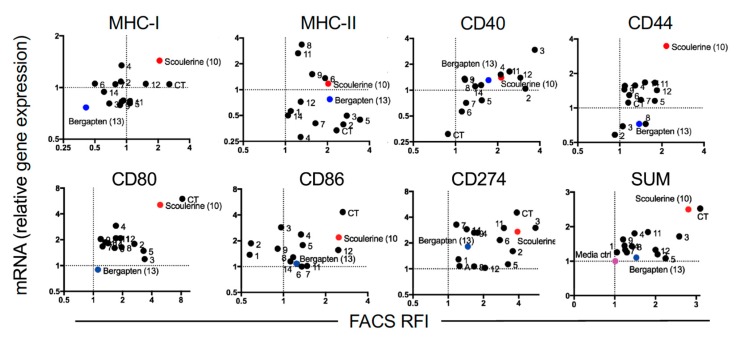
Correlation of modulation of mRNA and protein expression of immune molecules by plant compounds. The up-regulated and down-regulated gene expression induced by compounds falls in the upper right quadrant and the lower left quadrant, respectively. Scoulerine (**10**) and bergapten (**13**) are labeled red and blue circles, respectively. Other compounds tested are labeled as black circles. Culture media is labeled as a pink circle.

FACS-based assays give an excellent indication of protein expression, but they do not indicate whether gene expression has been modulated by the compound. Furthermore, possible aberrations associated with this particular case could include potential autofluorescence of the compound itself or cellular autofluorescence induced by the compound. Moreover, protein expression detected by FACS (and particularly surface expression) could be transiently modulated. Hence, measuring changes in gene expression is a very informative accompaniment to FACS-based assays. Accordingly, we co-cultured DC2.4 cells overnight with the most active concentration of the compound as determined above in the FACS-based assay (Table 2). The following day, cells were harvested and 20,000 live cells from each condition were sorted by FACS into RLT buffer and RNA extracted for mRNA analysis by RT-qPCR. The relative gene expression for each gene was calculated for each compound and compared to the media control. We then correlated gene expression with protein expression represented by the FACS RFI (Figure 3). This method allowed us to identify with confidence the compounds that upregulate expression of the genes of interest and the corresponding protein expression, which fall in the upper right quadrant of the mRNA gene expression versus FACS RFI plot. Conversely, compounds that down-regulate the gene and protein of interest fall in the lower left quadrant (Figure 3). The SUM of the changes for all genes and proteins relative to the media-only control is summarized in the bottom right corner of Figure 3 SUM.

Of the 14 compounds tested, scoulerine (**10**) stands out as a strong up-regulator of several important genes including *MHC-I*, *CD80* and *CD86*, suggesting that this compound would make a good potential adjuvant for a CD8^+^ T cell vaccine. Conversely, compound bergapten (**13**) is highlighted at the other end of the spectrum and it caused suppression of expression of some genes, and notably *MHC-I* at both the gene and protein level. Compound 14-*O*-acetylneoline (**3**) also showed moderate up-regulation of gene expression including *CD40* and *CD274* (*PDL-1*).

### 2.4. Cytotoxicity of Compounds with the Immortalized Non-Cancerous H69 Human Cholangiocyte Cell Line

**Figure 4:**
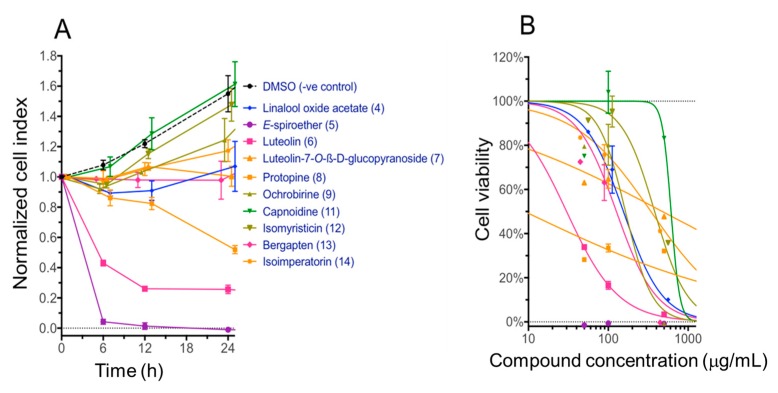
Cytotoxicity of selected plant compounds against immortalized H69 bile duct cells determined by xCELLigence system (ACEA Biosciences Inc.). (**A**) Cell toxicity over time as recorded by normalized cell index when treated with selected compounds. (**B**) Dose response toxicity curves at the 24 h time point show cell viability relative to the untreated control against concentration. Compounds **1**–**3** and **10** were not tested for cytotoxicity due to insufficient quantities available at the time of the study.

Of the 14 compounds tested in the DC assay, 10 of them: linalool oxide acetate (**4**), (*E*)-spiroether (**5**), luteolin (**6**), luteolin-7-*O*-β-d-glucopyranoside (**7**), protopine (**8**), ochrobirine (**9**), capnoidine (**11**), isomyristicin (**12**), bergapten (**13**), and isoimperatorin (**14**), were tested for cytotoxicity against normal bile duct H69 cells using an xCELLigence SP system (ACEA Biosciences Inc., San Diego, CA, USA) as described previously [28]. Quantities of compounds **1**, **2**, **3** and **10** were not sufficient to allow for cytotoxicity testing. Amongst the 10 compounds tested, compound **5** (*E*)-spiroether was the most cytotoxic with almost complete (96.5%) cell death from 6 h with 100 µg/mL and an IC_50_ value that was not able to be calculated with the concentrations assessed (<<<1 µg/mL) (Figure 4). Compound **6** (luteolin) was also reasonably toxic from 6–24 h and showed a 24 h IC_50_ value of 30 µg/mL (95% confidence interval 19–38 µg/mL). Protopine (**8**) showed minor (23%) cell toxicity from 6 h but that increased to 68% at 24 h, generating a more potent but highly variable IC_50_ value of 9 µg/mL (95% confidence interval 1–52 µg/mL).

Compound bergapten (**13**) that demonstrated interesting immunomodulatory activity in the DC assay showed far less potent cytotoxicity with the IC_50_ value of 126.4 µg/mL (95% confidence interval 76–248 µg/mL). This was consistent with moderate up-regulation of CD274 (Figure 3), which is the marker for Programmed Death Ligand 1 (PD-L1) known to play a key role in suppressing the immune system. Compound **10**, although not tested in this H69 cell system, has been reported by others to exhibit significant cytotoxic activity against lung carcinoma (A549) and HT-29 cancer cell lines that arrest the proliferation of ovarian carcinoma (A2780) and breast adenocarcinoma (SK-B-3 and MCF-7) cells [38], consistent with its potential adjuvant properties indicated in the DC cell assay.

## 3. Discussion

Medicinal plants have been an important source of modern drugs for many centuries and are receiving increasing attention worldwide with advancement in drug discovery techniques and technologies [39]. Plants produce various secondary metabolites that belong to different major phytochemical classes including flavonoids, tannins, terpenoids, saponins, triterpenoid saponins, alkaloids, phytosterols, carotenoids, fatty acids and essential oils, which are used for their defense and protection against predators/herbivores. Some of these metabolites display immunomodulatory properties including immunosuppression, immunostimulation and tolerogenicity through dynamic regulation of the target immune systems [40]. While immunostimulators are typically used for remedying infections, immunodeficiency and cancers, immunosuppressants are often used in organ transplantation and for treating autoimmune diseases. Tolerogens on the other hand are known for making the immune system non-responsive to targets [9]. The use of plant-derived secondary metabolites as novel drugs and immunomodulatory agents is a rapidly growing field of investigation [9]. The main categories of plant-derived secondary metabolites which have been transformed into modern drugs include terpenes (34%), glycosides (32%), alkaloids (16%) and others (18%) [41]. These drugs were discovered mostly using three strategies: bio-rational, chemo-rational and random approaches. One promising avenue of the bio-rational approach is ethno-medically directed screening based on the observation of pest-plant analysis, plant characteristics and their ecological adaptations and functions [39]. Among these three search strategies, the ethno-directed bio-rational approach has been proven to offer a higher hit rate of biological activities owing to a long clinical history of medicinal plants use by humans for treating various disorders [42]. Such evidence supports the need to investigate medicinal plant compounds for their usefulness as immunomodulatory agents or immunotherapies.

In the present study, based on the above rationale, we selected five medicinal plants—*A. laciniatum*, *A.* *nubigena*, *C. crispa*, *C. dubia,* and *P. amabile*—which are used in the scholarly Bhutanese traditional medicines for treating various disorders including fever, inflammation, malaria, tumor, leprosy, gout, mumps, abscess, liver and heart infections (see Table 1 for individual plant uses) [36]. Our initial phytochemical investigation of these plants for major chemotypes showed that their content was primarily alkaloids, terpenoids, tannins, and saponins and that their crude extracts possess antimalarial, antiparasitic, anti-inflammatory and antimicrobial properties [29,36]. To follow up on these findings, we have isolated 40 compounds belonging to different chemotypes (Table 1) using column and preparative thin layer chromatography. Their structures were determined using GC-MS and NMR as described earlier [31,32,33,34,35]. Of these, 14 major compounds were evaluated here for cytotoxicity and immunomodulatory properties using the xCELLigence system and a DC-based immunoassay, respectively. This DC-based immunoassay is an unbiased functional screen and a highly sensitive, high throughput–compatible screening platform for the discovery of DC-targeted small molecule immunomodulators with clinical applicability. Most of the currently known DC-stimulatory agents have been identified by testing rationally selected compounds for their in vitro capacity to induce phenotypic and functional changes that would be expected to accompany DC maturation or by testing a limited array of natural products for their in vivo capacities to augment protective immune responses against cancer or infectious microbes [43]. In this study, we combined FACS-based analysis of immune molecule expression with PCR-based assessment of gene expression. We used this method as we believe concordance in immune molecule regulation at the protein and gene level provides confidence in the actions of the compounds. Perhaps not surprisingly, we noted some discord between the relative effects on gene and protein expression for some compounds [44]. This may be due to several factors, including temporal differences between gene expression and protein expression, or spatial differences such as intracellular protein expression that is not accessible to surface staining by antibodies.

Of the 14 compounds tested here, scoulerine (**10**) strongly up-regulated several important genes and proteins including *MHC-I*, *CD80* and *CD86*, suggesting that this compound would potentially make a good immunologic adjuvant for a CD8^+^ T cell vaccine. Current vaccines intended for human use incorporate various adjuvants made from proteins, oligonucleotides, liposomes, complex natural products and drug-like small molecules [45]. Among the extant adjuvants, small molecule adjuvants (SMAs) are under-appreciated despite their versatile chemical structures and properties, which can be tailored to specific biological functions, targets and pathways. A review by Flower [45] described a number of SMAs derived from natural products as well as fully-synthetic drug-like molecules and identified as many as 15 natural products that SMAs isolated from plants, fungi, shark oil and marine bryozoans as peptides, lipids, triterpenes, glucosides, saponins, nucleotides, and macrolide lactones. For examples, QS-21 and Quil A are the purified saponin-based plant SMAs (derived from *Quillaja saponaria*) with defined mechanism of action, which are used as adjuvants in vaccines against HIV and cancer [46,47,48].

Since scoulerine (**10**) is an alkaloid, its mechanism of action may be novel and different to the saponin-based QS-21 and Quil A adjuvants, and therefore it merits further medicinal exploration. Scoulerine (**10**) has been reported from other plants including opium plant (*Croton flavens*) and other *Corydalis* species including *C. calliantha* and *C. cava* [49,50]. Notably, this compound possesses multiple important pharmacological properties. For example, it showed cytotoxicity against lung carcinoma (A549), ovarian carcinoma (A2780), breast adenocarcinoma (SK-B-3 and MCF-7) and gastrointestinal cancer cell lines [38]. It was identified as a potent antimalarial drug lead compound against multi-drug resistant *Plasmodium falciparum* [32]. This compound demonstrated antiemetic, antitussive, antibacterial, antimitotic, antiproliferative, proapoptotic and anti-Alzheimer’s activities [50,51]. Scoulerine (**10**) also protects α-adrenoreceptors against irreversible blockades caused by phenoxybenzamine, inhibits [^3^H]-inositol monophosphate formation caused by noradrenaline, acts as a selective α_1D_-adrenoreceptor antagonist without affecting the aorta contraction, and it has an affinity for the GABA receptors [52]. With such a diversity of therapeutic properties, scoulerine (**10**) is worthy of further exploration either as an independent drug entity or as an immunomodulator/adjuvant to potentiate other drugs/vaccines, including cancer chemotherapy, malaria vaccine development and Alzheimer’s disease.

At the other end of the spectrum, bergapten (**13**) consistently induced down-modulation of MHC-I and MH-II expression, indicative of anti-inflammatory and/or immunosuppressant activity while displaying low cytotoxicity. This compound belongs to the chemical class of furanocoumarins which are also produced by vegetables/herbs/fruits consumed by humans on a daily basis. Indeed, bergapten (**13**) has been used in the cosmetic and pharmaceutical industries for treating inflammatory skin diseases, such as atopic dermatitis, and pigment disorders like vitiligo and psoriasis, which is consistent with our findings about the ability of this compound to down-regulate DC activity [53,54,55]. In addition, bergapten (**13**) has demonstrated anti-proliferative activity in vitro against HL60 and A431 epithelial cancer cell lines [56] as well as human hepatocellular carcinoma cell line [57]. These reported activities are consistent with the immunosuppressive activity identified herein. To the best of our knowledge, the specific mechanism of action for DC-driven immunomodulation by these two compounds remains unreported. However, Habartova et al. [38] showed that the potent antiproliferative and pro-apoptotic function of scoulerine (**10**) in cancer cells occurs through its action to interfere with the microtubule elements of the cytoskeleton, checkpoint kinase signaling and p53 proteins. The mechanism of action of bergapten (**13**) against breast cancer cells has been illustrated by de Amicis et al. [58], which indicated that this compound drives autophagy through the upregulation of the oncosuppressor gene—phosphatase tension homologue (PTEN) expression.

With such a diversity of therapeutic properties, scoulerine (**10**) and bergapten (**13**) are worthy of further exploration either as an independent drug entity or as an immunomodulator/adjuvant to potentiate other drugs/vaccines, including cancer chemotherapy, malaria vaccines and Alzheimer’s disease. For a natural product-based drug discovery, isolating and obtaining enough quantities of the lead compound from natural sources is a big challenge and this has been identified as the bottleneck for advanced large-scale medicinal experimentations including lead optimization, pharmacokinetics and clinical trials. While the lead optimization comprises analogue development, total synthesis, rational drug design and quantitative structure-activity relationship (QSAR); the pharmacokinetics studies include adsorption, distribution, metabolism, excretion and toxicity (ADMET) evaluations. Pharmacodynamics study, which is seldom grouped with pharmacokinetics, provides information on what the drug does to the body and would include dosage formulations. All these drug development studies require large supplies of the drug lead compound and therefore laboratory synthesis of such a compound is very critical. Total synthesis or analogue development of the lead compound could take a synthetic chemist between 1–4 years depending upon the nature and the complexity of the structures. Synthetic chemistry of natural products is often plagued by low success rate of compound synthesis. Even if the compound is successfully synthesized, the synthetic steps may be lengthy, expensive or may afford minute amounts only. Interestingly, both scoulerine (**10**) and bergapten (**13**) had been successfully and efficiently synthesized in the laboratory using a biocatalytic synthetic method and the photochemical aromatic annulation strategy, respectively [59,60]. These make the two new drug lead compounds even more appealing for drug development.

## 4. Materials and Methods

### 4.1. Collection and Extraction of Medicinal Plants

As described previously [31,32,33,34,35], we have selected five medicinal plants for this study and their traditional uses are provided in Table 1. The root of *A. laciniatum* (Ranunculaceae) was collected from the opposite site of Lingshi Makhang (altitude: 4183 m; latitude: 27°50′29.9″; longitude: 89°25′41.5″; global positioning system point number (GPSPN): 138; site number: P138; Slope: 25°; aspect: North-East) and was assigned herbarium voucher specimen number (HVSN) 93. The aerial components of *A. nubegina* (Compositae) were collected from Lingzhi and were assigned as HVSN 73. The whole plant of *C. crispa* (Fumariaceae) was collected from Thuphu (altitude: 3962 m; latitude: 27°51′15.4″; longitude: 89°27′12.8″; GPSPN: 187; site number: P187; slope: 40°; aspect: South-East) and was assigned as HVSN 78. The whole plant of *C. dubia* (Fumariaceae) was collected from Thruenchela (altitude: 4651 m; latitude: 27°56′00.1″; longitude: 89°26′11.6″; GPSPN: 167; site number: P167; slope: 30°; aspect: North-West) and was assigned as HVSN 78. The aerial parts of *P. amabile* (Umbelliferae) were collected from Lingzhi (4200 m) and were assigned as HVSN 29. All herbarium specimens were deposited at the Pharmaceutical and Research Unit, Ministry of Health in Bhutan. The dried plant material (2 kg) was chopped and made into powder form and was repeatedly extracted with analytical grade or HPLC grade methanol (5 × 3 L over 48 h). The extract was filtered and then concentrated using a rotary evaporator at 35–50 °C to afford the crude methanol extract of each medicinal plant.

### 4.2. Isolation and Preparation of Compounds for In Vitro Screening Assays

Since the five target plants contain different phytochemicals, we used two main types of natural product isolation methods as described by us previously [39]. For alkaloid-containing medicinal plants including *A. laciniatum*, *C. crispa* and *C. dubia*, we first used an acid-base fraction method to obtain the total alkaloids present in their MeOH extracts. The MeOH extract of each plant was acidified with HCl (5%) and fractionated successively using hexane (5 × 60 mL) and CH_2_Cl_2_ (5 × 60 mL) to yield hexane and dichloromethane extracts, respectively. The aqueous solution was then basified (pH 9–11) with NH_4_OH solution and fractionated with CHCl_3_ (5 × 60 mL) to obtain a chloroform extract, which was rich in total alkaloids. Focusing on alkaloids, these CHCl_3_ extracts were repeatedly separated using flash column chromatography (packed with Merck Kieselgel 60 PF254 (Merck, French Forest, Australia)) and pre-coated silica plates (0.2 mm silica thickness, Merck). Finally, a total of 22 pure alkaloids were obtained from *A. laciniatum*, *C. crispa* and *C. dubia* as amorphous solids or crystals [31,32,35]. Seven major alkaloids were selected for testing in the DC and cytotoxicity bioassays. Specifically, five alkaloids were isolated from *A. laciniatum*, and three of them were selected for bioactivity screening: pseudaconitine (**1**), 14-veratryolpseudaconitine (**2**) and 14-*O*-acetylneoline (**3**). From *C. crispa*, nine alkaloids were isolated and two major compounds—protopine (**8**) and ochrobirine (**9**)—were selected for bioactivity screening. Eight alkaloids were isolated from the related species, *C. dubia*, and two of them—scoulerine (**10**) and capnoidine (**11**)—were selected for DC and cytotoxicity studies.

For non-alkaloidal medicinal plants including *A. nubigena*, we used a solvent-based polarity fractionation method [33]. First, we fractionated the extract using hexane, followed by ethyl acetate, to yield hexane and ethyl acetate extracts, respectively. Repeated separation of ethyl acetate extract using column and preparative thin layer chromatography (Merck, Aluminium backing) resulted in the isolation of seven compounds (Table 1). Four of the seven compounds were selected for the bioactivity assays: linalool oxide acetate (**4**), (*E*)-spiroether (**5**), luteolin (**6**), and luteolin-7-*O*-β-d-glucopyranoside (**7**) (Figure 1). For the isolation of phenylpropanoids and furanocoumarins from *P. amabile*, as reported earlier [34], we dissolved the crude MeOH extract in MeOH/water (1:9) and first fractionated with hexane followed by petroleum spirit. The aqueous portion was acidified with HCl (5%), fractionated with CH_2_Cl_2_, basified with NH_4_OH (at pH 9–12) and then finally fractionated with CH_2_Cl_2_ to generate the basified CH_2_Cl_2_ extract. This basified CH_2_Cl_2_ extract, upon repeated purification using the chromatographic methods described above, yielded 10 compounds (Table 1). Of these 10 compounds, three compounds—isomyristicin (**12**), bergapten (**13**), and isoimperatorin (**14**) (Figure 1)—were obtained in sufficient quantities to carry out the DC and cytotoxicity assays.

The instrumentation for phytochemical identification and structure elucidation were conducted as reported earlier by us [31,32,33,34,35]. Briefly, for determining physiochemical properties including melting point and optical rotation values, we used a Reichert hot-stage apparatus and a JASCO 2000 Series polarimeter, respectively. An average of 10 optical readings were taken to obtain the observed rotation value. For determining the functional group of a compound, we used a Smart Omni-Sampler Avator ESP Nicolet spectrometer. For obtaining the LR-ESI-MS mass and the LR-EI-MS mass of a compound, we used a Micromass Waters Platform LCZ (single quadrupole, MeOH as solvent) and a Shimadzu GCMS-QP-5050 (DI at 70 eV), respectively. A Micromass Waters Q-ToF Ultima (quadrupole time-of-flight) mass spectrometer was used for acquiring the HR-ESI-MS-based molecular formula. GC-MS used NIST and NISTREP mass spectra libraries of GC-MS data for comparing the mass spectra of the plant compounds. For obtaining NMR spectra (^1^H-NMR, gCOSY, ^13^C-NMR, APT, gHMBC, gHSQC, and gNOESY, deuterated solvents—CD_3_OD or CDCl_3_) and structure elucidation of a compound, we used a 500 MHz Varian Unity Inova, 500 MHz Varian Premium Shield (VNMRS PS 54), and 300 MHz Varian Mercury spectrometer.

Wherever crystals were obtained for a compound, crystal structures were determined using x-Ray crystallography (PLATON program). Hydrogen atoms were included at calculated positions and were initially refined to regularize their geometry (C–H in the range 0.93–0.98Å) and on U_iso_(H) (in the range 1.2–1.5 times U_eg_ of the parent atom). Computing details include data collection: COLLECT; cell refinement and data reduction: DENZO/SCALEPACK; program (s) used to solve structure: USER DEFINED STRUCTURE SOLUTION; molecular graphics: ORTEP-II in TEXSAN; and program(s) used to refine structure and prepare material for publication: CRYSTALS [61]. The X-ray diffraction images were measured on a Nonius KappaCCD diffractometer (Mo *Kθ* radiation, graphite monochromator, *θ* = 0.71073 Å), data were extracted using the DENZO package and by direct methods (SUPERFLIP, SIR92) [62]. The structures were refined using the CRYSTALS program package. Atomic coordinates, bond lengths and angles and displacement parameters have been deposited at the Cambridge Crystallographic Data Centre (CCDC no. 929725, 929726). These data can be obtained from https://www.ccdc.cam.ac.uk/ or by emailing data_request@ccdc.cam.ac.uk, or by contacting The Cambridge Crystallographic Data Centre, 12 Union Road, Cambridge CB2 1EZ, UK; Fax: +44-1223-336033.

Detailed characterization of each compound using the above equipment and techniques generated the data that shows the level of their purity and distinguishes them from each other as follows.

Pseudaconitine (**1**): Prismic crystals (1.9 g, from CHCl_3_/MeOH, (1:1)). LR-ESI-MS (*m*/*z*): 690 [M + H^+^]. LR-EI-MS (*m*/*z*): 689 [M^+^], 675, 658, 642, 629, 614, 598, 585, 464, 432, 330, 266, 252, 236, 202, 182, 178, 165, 137, 86, 75, 58, 45. ^1^H NMR (500 MHz, CDCl_3_): δ 3.04–3.20 (1H, m, H-1), 1.95–2.11 (1H, m, H_a_-2), 2.25–2.45 (1H, m, H_b_-2), 3.79 (1H, m, H-3), 2.10–2.16 (1H, m, H-5), 4.03 (1H, d, *J* = 6.5 Hz, H-6), 2.90 (1H, m, H-7), 2.89–2.95 (1H, m, H-9), 2.10–2.15 (1H, m, H-10), 2.55–2.63 (2H, m, H-12), 4.87 (1H, d, *J* = 4.5 Hz, H-14), 2.40–2.55 (1H, m, H_a_-15), 3.06–3.14 (1H, m, H_b_-15), 3.38–3.41 (1H, t, *J* = 7.5, H-16), 3.02 (1H, br s, H-17), 3.51–3.53 (1H, m, H_a_-18), 3.62–3.65 (1H, m, H_b_-18), 2.25–2.45 (1H, m, H_a_-19), 2.90–2.96 (1H, m, H_b_-19), 2.35–2.60 (2H, m, H-20), 1.13 (3H, t, *J* = 7.0 Hz, 21-Me), 3.26 (3H, s, 1-OMe), 3.17 (3H, s, 6-OMe), 3.54 (3H, s, 16-OMe), 3.30 (3H, s, 18-OMe), 3.91 (3H, s, 3′-OMe), 3.94 (3H, s, 4′-OMe), 1.34 (3H, s, 8-OAc), 2.10 (1H, s, 3-OH), 3.85 (1H, s, 13-OH), 7.62 (1H, s, H-2′), 6.90 (1H, d, *J* = 8.5, H-5′), 7.70 (1H, d, *J* = 8.0, H-6′).

14-Veratroylpseudaconine (**2**): Amorphous white solid (28.7 mg). LR-ESI-MS (*m*/*z*): 648 [M + H^+^]. LR-EI-MS (*m*/*z*): 647 [M^+^], 631, 616, 599, 557, 435, 182, 165, 149, 125, 111, 97, 71, 57, 41. HR-ESI-MS (molecular formula): C_34_H_49_NO_11_. ^1^H NMR (500 MHz, CDCl_3_): δ 3.13 (1H, dd, *J* = 6.5 Hz, H-1), 2.04–2.11 (1H, m, H_a_-2), 2.26–2.46 (1H, m, H_b_-2), 3.64–3.77 (1H, m, H-3), 2.04–2.11 (1H, m, H-5), 4.07 (1H, d, *J* = 6.5 Hz, H-6), 2.46–2.52 (1H, m, H-7), 2.52–2.57 (1H, m, H-9), 2.04–2.11 (1H, m, H-10), 2.03–2.11 (1H, m, H_a_-12), 2.31–2.57 (1H, m, H_b_-12), 5.12 (1H, d, *J* = 5.5 Hz, H-14), 2.27–2.41 (1H, m, H_a_-15), 2.51–2.64 (1H, m, H_b_-15), 3.33–3.38 (1H, m, H-16), 3.01 (1H, br s, H-17), 3.64–3.77 (2H, m, H-18), 2.43–2.45 (1H, m, H_a_-19), 2.88–3.07 (1H, m, H_b_-19), 2.39–2.57 (2H, m, H-20), 1.11 (3H, t, *J* = 7.0 Hz, 21-Me), 3.28 (3H, s, 1-OMe), 3.25 (3H, s, 6-OMe), 3.42 (3H, s, 16-OMe), 3.31 (3H, s, 18-OMe), 3.93 (3H, s, 3′-OMe), 3.94 (3H, s, 4′-OMe), 2.06 (1H, br s, 3-OH), 2.27 (1H, s, 8-OH), 3.67 (1H, s, 13-OH), 7.60 (1H, br s, H-2′), 6.90 (1H, d, *J* = 8.5, H-5′), 7.67 (1H, d, *J* = 8.5, H-6′). ^13^C NMR (125 MHz, CDCl_3_): δ 82.7 (C-1), 33.7 (C-2), 72.1 (C-4), 42.5 (C-4), 48.0 (C-5), 82.6 (C-6), 53.6 (C-7), 74.0 (C-8), 47.9 (C-9), 42.1 (C-10), 50.4 (C-11), 35.8 (C-12), 76.0 (C-13), 79.9 (C-14), 42.2 (C-15), 83.3 (C-16), 62.0 (C-17), 77.5 (C-18), 47.6 (C-19), 49.1 (C-20), 13.6 (C-21), 56.0 (C-1′), 57.7 (C-6′), 58.5 (C-16′), 59.3 (C-18′), 166.5 (O = C), 122.5 (C-1), 110.5 (C-2), 148.8 (C-3), 153.3 (C-4), 112.3 (C-5), 123.9 (C-6), 56.2 (C-3′), 56.0 (C-4′).

14-*O*-Acetylneoline (**3**): Amorphous white solid (46.9 mg). LR-ESI-MS (*m*/*z*): 690 [M + H^+^]. HR-EI-MS (molecular formula): C_26_H_41_NO_7_. ^1^H NMR (500 MHz, CDCl_3_): δ 3.69 (1H, br s, H-1), 1.50–1.63 (2H, m, H-2), 1.57–1.63 (2H, m, H-3), 2.19 (1H, d, *J* = 7.0, H-5), 4.11 (1H, d, *J* = 6.0 Hz, H-6), 2.00 (1H, s, H-7), 2.24 (1H, t, *J* = 5.5 Hz, H-9), 2.60–2.63 (1H, m, H-10), 1.77–1.81 (2H, m, H-12), 2.30–2.35 (1H, m, H-13), 4.85 (1H, t, *J* = 4.0 Hz, H-14), 1.88–1.93 (1H, m, H_a_-15), 2.30–2.32 (1H, m, H_b_-15), 3.29–3.30 (1H, m, H-16), 2.67 (1H, s, H-17), 3.23–3.62 (2H, d, *J* = 8.0 Hz, H-18), 2.31–2.69 (2H, m, H-19), 2.45–2.58 (2H, m, H-20), 1.13 (3H, t, *J* = 7.0 Hz, 21-Me), 3.34 (3H, s, 6-OMe), 3.26 (3H, s, 16-OMe), 3.32 (3H, s, 18-OMe), 2.06 (3H, s, OCO-Me). ^13^C NMR (125 MHz, CDCl_3_): δ 72.2 (C-1), 29.4 (C-2), 30.0 (C-4), 30.2 (C-4), 44.5 (C-5), 83.4 (C-6), 52.7(C-7), 74.7 (C-8), 46.2 (C-9), 36.7 (C-10), 49.7 (C-11), 29.6 (C-12), 43.4 (C-13), 77.2 (C-14), 42.7 (C-15), 82.0 (C-16), 63.4 (C-17), 80.2 (C-18), 57.1 (C-19), 48.4 (C-20), 13.1 (C-21), 58.1 (6-OMe), 56.2 (16-OMe), 59.3 (18-OMe), 21.3 (OCO-Me), 170.5 (O-C-OMe).

Linalool oxide acetate (**4**): Colourless oil (1.2 g). LR-ESI-MS (*m*/*z*): 213 [M + H^+^]. LR-EI-MS (*m*/*z*): 212 (100%), 197, 195, 154, 153, 136, 114, 101, 94, 79, 68, 43. ^1^H-NMR (CDCl_3_, 500 MHz): δ 4.63 (1H, m, H-3), 2.16–2.09 (2H, m, H-4), 1.59–1.83 (2H, m, H-5), 5.92–6.02 (1H, m, H-7), 4.96–5.02 (2H, m, H-8), 1.17 (3H, s, H-9), 1.15 (3H, s, H-10), 1.20 (3H, s, H-11), 2.02 (3H, s, H-13). ^13^C-NMR (125 MHz, CDCl_3_): δ 74.1 (C-2), 76.0 (C-3), 31.8 (C-4), 22.2 (C-5), 73.5 (C-6), 146.0 (C-7), 110.7 (C-8), 31.3 (C-9), 21.9 (C-10), 29.3 (C-11), 170.4 (C-12), 21.2 (C-13).

(*E*)-spiroether (**5**): Colourless liquid (87.0 mg). LR-ESI-MS (*m*/*z*): 201 [M + H^+^]. LR-EI-MS (*m*/*z*): 200 (M^+^) (100%), 185, 170, 157, 141, 128, 115, 102, 76. ^1^H-NMR (CDCl_3_, 500 MHz): δ 1.98 (3H, s, H-1), 4.92 (1H, br s, H-6), 6.69 (1H, d, *J* = 5.7, H-8), 6.21 (1H, t, *J* = 5.0, H-9), 2.00–2.30 (4H, m, H-11/12), 3.87–4.20 (2H, m, H-13). ^13^C-NMR (125 MHz, CDCl_3_, APT): δ 4.9 (C-1), 80.8 (C-2), 65.4 (C-3), 79.1 (C-4), 70.9 (C-5), 79.0 (C-6), 167.3 (C-7), 127.6 (C-8), 135.4 (C-9), 121.2 (C-10), 35.8 (C-11), 24.7 (C-12), 69.9 (C-13).

*Luteolin (**6**)*: White solid (618.0 mg). LR-ESI-MS (*m/z*): 281 [M+H^+^]. LR-EI-MS (*m/z*): 286 (M^+^), 277, 258, 229, 153 (100%), 124, 96, 77, 69. ^1^H NMR (500 MHz, CD_3_OD): δ 6.19 (1H, br s), 6.41 (1H, br s), 6.51 (1H, br s), 6.88 (1H, d, *J* = 9.0 Hz), 7.35 (1H, s), 7.36 (1H, s). ^13^C NMR (125 MHz, CD_3_OD): δ 183.8 (C-4), 166.3 (C-2), 166.1 (C-7), 163.2 (C-5), 159.4 (C-9), 150.9 (C-4), 147.1 (C-3), 123.6 (C-6), 120.2 (C-1), 116.7 (C-5), 114.1 (C-2), 103.8 (C-10), 103.8 (C-3), 100.1 (C-6), 94.9 (C-8).

Luteolin-7-*O*-β-d-glucopyranoside (**7**): Faint yellow powder (41.3 mg). LR-ESI-MS (*m*/*z*): 449 [M + H^+^]. LR-EI-MS (*m*/*z*): 448, 281, 207, 191, 147, 133, 84, 73, 66, 44. ^1^H NMR (500 MHz, DMSO): δ 7.44 (1H, dd, *J* = 8.5, 2.5 Hz, H-6′), 7.40 (1H, s, H-2′), 6.88 (1H, d, *J* = 8.0 Hz, H-5′), 6.77 (1H, d, *J* = 2.0 Hz, H-8), 6.74 (1H, s, H-3), 6.43 (1H, d, *J* = 2.0 Hz, H-6), 5.07 (1H, d, *J* = 7.0 Hz, glc-1), 3.14–3.70 (6H, m, glc-2 to glc-6). ^13^C NMR (125 MHz, DMSO): δ 181.9 (C-4), 164.5 (C-2), 162.9 (C-7), 161.1 (C-5), 156.9 (C-9), 150.0 (C-4′), 145.8 (C-3′), 121.3 (C-1′), 119.1 (C-6′), 115.9 (C-5′), 113.5 (C-2′), 105.3 (C-10), 103.1 (C-3), 99.8 (glc-1), 99.5 (C-6), 94.7 (C-8), 77.1 (glc-5), 76.4 (glc-3), 73.1 (glc-2), 69.5 (glc-4), 60.6 (glc-6).

Protopine (**8**): Prisms (MeOH/CHCl_3_, 1.0 g). mp: 209–211 °C. Optically inactive. LR-ESI-MS (*m*/*z*) 354 [M + H^+^]. LR-EI-MS (*m*/*z*): 353 [M^+^], 295, 281, 267, 251, 237, 223, 209, 190, 177, 163, 148 (100%), 134 and this ion fragmentation pattern matched that of protopine reported in the MS library (NIST08s, Entry # 26245, CAS: 130-86-9, RetIndex: 2943). HR-ESI-MS (molecular formula): C_20_H_19_NO_5_. ^1^H-NMR (500 MHz, CDCl_3_): δ 6.90 (1H, s, H-1), 6.64 (1H, s, H-4), 2.65 (2H, br s, H-5), 2.59 (2H, br s, H-6), 3.61 (2H, br s, H-8), 6.68 (1H, d, *J* = 7.5 Hz, H-11), 6.66 (1H, d, *J* = 8.0 Hz, H-12), 3.75 (2H, br s, H-13), 5.94 (2H, s, 2,3-OCH_2_O), 5.92 (2H, s, 9,10-OCH_2_O), 1.96 (3H, s, 7-NCH_3_). ^13^C-NMR (125 MHz, CDCl_3_): δ 108.0 (C-1), 145.9 (C-2), 148.0 (C-3), 110.3 (C-4), 132.7 (C-4′), 31.5 (C-5), 57.6 (C-6), 50.9 (C-8), 117.5 (C-8′), 146.2 (C-9), 146.0 (C-10), 106.8 (C-11), 124.9 (C-12), 128.7 (C-12′), 46.1 (C-13), 194.3 (C-14), 136.0 (C-14′), 101.2 (2,3-OCH_2_O), 41.5 (7-N-CH_3_), 100.9 (9,10-OCH_2_O).

Ochrobirine (**9**): Prisms (MeOH/CHCl_3_, (60.6 mg)); mp: 203–207 °C. [α]_D_^25^ + 38.7° (*c* 0.36, CHCl_3_). LR-ESI-MS (*m*/*z*): 370 [M + H^+^]. LR-EI-MS (*m*/*z*): 369 [M^+^], 351, 336, 322 (100%), 293, 264, 204, 190 and this ion fragmentation pattern matched that of ochrobirine in the MS library (NIST08.LIB, Entry # 154984, CAS: 24181-64-4, RetIndex: 3185). HR-ESI-MS (molecular formula): C_20_H_19_NO_6_. ^1^H-NMR (500 MHz, CDCl_3_): δ 6.85 (1H, s, H-1), 6.64 (1H, s, H-4), 2.54 (2H, br s, H-5), 3.25 (2H, br s, H-6), 4.87 (2H, s, H-8), 6.03 (1H, s, H-11), 6.87 (1H, s, H-12), 5.42 (2H, d, *J* = 10.5 Hz, H-13), 2.66 (3H, s, N-CH_3_), 5.83 (2H, s, 2,3-OCH_2_O), 6.01 (2H, s, 9,10-OCH_2_O). ^13^C-NMR (125 MHz, CDCl_3_): δ 109.4 (C-1), 145.8 (C-2), 146.3 (C-3), 109.8 (C-4), 126.0 (C-4′), 22.5 (C-5), δ 47.4 (C-6), 73.7 (C-8), 121.0 (C-8′), 144.2 (C-9), 148.3 (C-10), 106.7 (C-11), 116.0 (C-12), 140.0 (C-12′), 79.5 (C-13), 75.0 (C-14), 129.5 (C-14′), 37.5 (N-CH_3_), 100.8 (2,3-OCH_2_O), 101.6 (9,10-OCH_2_O).

Capnoidine (**10**): Orthorhombic crystal (80.3 mg). LR-ESI-MS (*m*/*z*): 368 [M + H^+^]. LR-EI-MS (*m*/*z*): 367 [M^+^], 207, 190 (100%), 175, 160, 149, 131, 117, 103 and this ion fragmentation pattern matched that of capnoidine reported in the MS library (NIST08.LIB, Entry # 153894, CAS: 485-49-4, RetIndex: 3142. HR-ESI-MS (molecular formula): C_20_H_17_NO_6_. ^1^H-NMR (500 MHz, CDCl_3_): δ 4.02 (1H, d, *J* = 3.0 Hz, H-1), 3.05–3.07 (2H, m, H-3), δ 2.44–2.74 (1H, m, H-4), 6.40 (1H, s, H-5), 6.67 (1H, s, H-8), 5.62 (1H, d, *J* = 3.0 Hz, H-9), 6.94 (1H, d, *J* = 7.5 Hz, H-10), 7.14 (1H, d, *J* = 8.0 Hz, H-11), 2.53 (3H, s, N-CH_3_), 5.84 (2H, s, OCH_2_O), 6.10 (2H, s, OCH_2_O). ^13^C-NMR (125 MHz, CDCl_3_): δ 66.2 (C-1), 51.4 (C-3), 29.2 (C-4), 125.2 (C-4a), 108.2 (C-5), 146.4 (C-6), 146.0 (C-7), 107.6 (C-8), 130.1 (C-8a), 82.9 (C-9), 140.9 (C-9a), 116.0 (C-10), 113.0 (C-11), 148.9 (12), 144.2 (C-13), 110.0 (C-13a), 45.1 (N-CH_3_), 100.9 (OCH_2_O) 103.2 (OCH_2_O), 167.5 (C = O).

Scoulerine (**11**): Reddish brown solid (9.4 mg). LR-EI-MS: *m/z* 327 [M^+^], 326, 312, 178 (100%), 176, 163, 150, 150, 135, 121 and 107. HR-EIMS (molecular formula): C_19_H_21_NO_4_. ^1^H-NMR (CDCl_3_, 500 MHz): δ 6.82 (1H, s H-1), 6.59 (1H, s, H-4), 2.67–3.14 (2H, m, H-5), 2.61–3.19 (2H, m, H-6), 3.48 (1H, d, *J* = 15.5 Hz, H_a_-8) and 4.24 (1H, d, *J* = 15.5 Hz, H_b_-8), 6.72 (1H, d, *J* = 8.0 Hz, H-11), 6.67 (1H, d, *J* = 8.0 Hz, H-12), 2.78–2.84 (1H, m, H_a_-13) and 3.25 (1H, d, *J* = 16.5, H_b_-13), 3.54 (1H, br s, H-13a), 3.86 (3H, s, 3-MeO), 3.87 (3H, s, 10-MeO). ^13^C-NMR (125 MHz, CDCl_3_): δ 109.1 (C-1), 145.1 (C-2), 144.1 (C-3), 111.5 (C-4), 130.8 (C-4a), 29.3 (C-5), 51.7 (C-6), 53.6 (C-8), 121.3 (C-8a), 141.6 (C-9), 144.0 (C-10), 110.8 (C-11), 119.5 (C-12), 128.3 (C-12a), 36.4 (C-13), 59.3 (C-13a), 126.2 (C-13b), 56.3 (3-OCH_3_), 56.2 (10-OCH_3_).

*Isomyristicin (**12**)*: Colourless oil (185.3 mg). LR-ESI-MS (*m/z*): 193 [M+H^+^]. LR-EI-MS (*m/z*): 192 (100%), 177, 165, 161, 147, 131, 119, 103, 91, 77, 65, 53, 39. ^1^H NMR (500 MHz, CDCl_3_): δ 6.45 (1H, s, H-2), 6.55 (1H, s, H-6), 6.29 (1H, d, *J* = 15.6 Hz, H-1′), 6.07 (1H, m, H-2′), 1.84 (3H, d, *J* = 6.6 Hz, H-3′), 5.91 (2H, s), 3.87 (3H, s, OMe). ^13^C NMR (125 MHz, CDCl_3_): δ 133.2 (C-1), 99.7 (C-2), 143.7 (C-3), 134.5 (C-4), 149.3 (C-5), 106.2 (C-6), 131.0 (C-1′), 124.8 (C-2′), 18.6 (C-3′), 101.5 (OCH_2_O), 56.7 (OMe).

Bergapten (**13**): Clear crystals (2.5 g, from MeOH/CHCl_3_ (1:1)). LR-ESI-MS (*m*/*z*): 217 [M + H^+^]. LR-EI-MS (*m*/*z*): 216 [M^+^], 202 (100%), 192, 173, 158, 145, 131, 118, 102, 89, 74, 69, 63, 51. ^1^H NMR (500 MHz, CDCl_3_): δ 6.27 (1H, d, *J* = 9.5 Hz, H-3), 8.15 (1H, d, *J* = 10.0 Hz, H-4), 7.12 (1H, s, H-8), 7.59 (1H, d, *J* = 2.5 Hz, H-2′), 7.02 (1H, d, *J* = 2.0 Hz, H-3′), 4.27 (3H, s, 5-OMe). ^13^C NMR (125 MHz, CDCl_3_): δ 161.4 (C-2), 112.7 (C-3), 139.4 (C-4), 106.6 (C-4a), 149.7 (C-5), 112.9 (C-6), 158.5 (C-7), 93.9 (C-8), 152.9 (C-8a), 144.9 (C-2′), 105.2 (C-3′), 60.3 (5-OMe).

Isoimperatorin (**14**): Clear crystals (143.1 mg, from MeOH/CHCl_3_ (1:3)). LR-ESI-MS (*m*/*z*): 271 [M + H^+^]. LR-EI-MS (*m*/*z*): 270 [M^+^], 202 (100%), 174, 158, 145, 131, 118, 103, 89, 69, 51. ^1^H NMR (500 MHz, CDCl_3_): δ 6.26 (1H, d, *J* = 10.0 Hz, H-3), 8.15 (1H, d, *J* = 9.5 Hz, H-4), 7.14 (1H, s, H-8), 7.59 (1H, d, *J* = 2.0, Hz, H-2′), 6.95 (1H, d, *J* = 1.0 Hz, H-3′), 4.91 (2H, d, *J* = 7.5 Hz, H-1″), 5.53 (1H, t, *J* = 7.0 Hz, H-2″), 1.80 (3H, s, 4″-Me), 1.70 (3H, s, 5″-Me). ^13^C NMR (125 MHz, CDCl_3_): δ 161.4 (C-2), 112.7 (C-3), 139.6 (C-4), 107.6 (C-4a), 149.1 (C-5), 114.3 (C-6), 158.1 (C-7), 94.3 (C-8), 152.6 (C-9), 145.0 (C-2′), 105.2 (C-3′), 69.9 (C-1″), 119.3 (C-2″), 139.8 (C-3″), 25.9 (C-4″), 18.4 (C-5″).

For immunoregulatory screening, we selected 14 compounds based on their abundance or the quality isolated from each plant (minor compounds were not tested here). The stock solutions of the 14 compounds selected for cytotoxicity and immunoassays were prepared by initially dissolving 1 mg of weighed compounds in 10–20 µL of DMSO and then subsequently diluting them with 980–990 µL of relevant culture media to make the stock concentrations of 1 mg/mL.

### 4.3. DC Assay Method and Flow Cytometry

DC2.4 cells was a gift of Kenneth L. Rock, University of Massachusetts Medical School and Dana-Farber Cancer Institute (Boston, MA, USA). The DC2.4 cell line was established from bone marrow cells of C57BL/6 mice transduced with murine granulocyte macrophage-colony stimulating factor and retrovirally transfected with *raf* and *myc* oncogenes [25]. DC2.4 cells (4000 cells in exponential phase/200 µL) were cultured in 96-well flat bottom plates overnight with a titration of the test compound in media (stock concentration of 1 mg/mL and further titrated in tissue culture media at 4 mid-log dilutions starting at 100 µg/mL) or media only (modified DMEM, 50 µM 2-mercaptoethanol and 216 mg/L l-glutamine, 10% heat-inactivated FCS). Each compound was tested twice, in replicate assays. The cells were then harvested with trypsin, split in two and transferred to 96-well V-bottom plates, washed and stained on ice with FC-blocking antibody (2.4G2 supernatant) followed by combinations of fluorochrome-conjugated antibodies (all Biolegend except MHC-II) in two panels: (1) MHC-I FITC (H-2K^b^), CD86 AF700, MHC-II V500 (BD); (2) CD40 AF647, CD80 FITC, CD44 PeCy7, CD274 BV421 for 10 min on ice. Samples were then washed and resuspended in buffer with sytox blue (panel 1) or propidium iodide (panel 2) for dead cell exclusion and run on a LSRFortessa flow cytometer (BD Biosciences, San Jose, CA, USA). Post-acquisition data analysis was performed with FlowJo software version 9.1 (Treestar, Ashland, OR, USA); calculations were performed using Microsoft Excel (version 12, Microsoft Corporation, Washington, NM, USA). The RFI (relative fluorescence intensity) for each parameter was calculated by dividing the median fluorescence intensity (MFI) of the test sample by that of the control (media-only) samples.

### 4.4. Gene Expression Analysis

DC2.4 cells were co-cultured overnight with test compounds as described above. The following day, cells were harvested and 20,000 live cells from each condition were sorted by FACS into RLT buffer and stored at −70 °C. On the day of extraction, frozen cell lysates were thawed quickly on ice and mRNA was extracted using the RNEasy micro kit (Qiagen, Chadstone Centre, VIC, Australia) according to the manufacturer’s instructions. cDNA was synthesized using oligo-dT and AMVRT (Promega, Sydney, Australia) according to the manufacturer’s instructions. Gene expression was measured using individual Taqman gene expression assays (Applied Biosystems, Foster City, CA, USA) and Platinum Taq polymerase (Life Technologies, Carlsbad, CA, USA) in a 15 μL volume reaction using a Rotorgene 3000 PCR machine (Corbett Research, Mortlake, Australia) with the following conditions: 2 min at 50 °C for calibration of fluorescence gain values, then denaturing at for 2 min at 95 °C, followed by 45 cycles of 5 s at 95 °C and 30 s at 60 °C. Gene expression was quantified relative to a standard curve generated from a titration of cloned cDNA and relative gene expression was calculated by dividing the expression value of the test sample by that of the cells grown in media only. All assays were conducted in triplicate.

### 4.5. Determining Cytotoxicity Using xCELLigence RTCA System

The immortalized non-malignant human cholangiocyte cell line H69 is a SV40-transformed human bile duct epithelial cell line originally derived from a normal liver harvested for transplantation [63,64]. H69s were grown under similar conditions with growth factor-supplemented specialized complete media; DMEM/F12 with high glucose, 10% fetal bovine serum (FBS), 1× antibiotic/antimycotic, 25 µg/mL adenine, 5 µg/mL insulin, 1 µg/mL epinephrine, 8.3 µg/mL holo-transferrin, 0.62 µg/mL, hydrocortisone, 13.6 ng/mL T3 and 10 ng/mL EGF (Life Technologies). Cytotoxicity screening of the test compounds against the H69 cell line was performed using an xCELLigence SP system (ACEA Biosciences Inc., San Diego, CA, USA) as described by us previously [65]. All experiments were carried out as per the manufacturer’s instructions with 0.25–60 min read intervals using the real time cell assay (RTCA) software (ACEA Biosciences Inc., USA). All assays were conducted in triplicate. Inter-well spaces were filled with 100 µL of culture medium or PBS to prevent evaporation. The E-plates containing cells were seeded at 5000 cells/well and incubated overnight at 37 °C with 5% CO_2_. The cells were then treated with prepared concentrations (50, 100, 500 µg/mL) of the test compounds and the viability of the cells was monitored continually for 24 h.

### 4.6. IC_50_ Calculations of Cytotoxicity

We determined the IC_50_ values of test compounds based on the normalised cell index (nCI) for cells, as described previously [66]. Briefly, the 24 h nCI relative to the negative DMSO treated controls (deemed 100%) on the *y*-axis was plotted against the log concentration value *x*-axis. GraphPad Prism 6.0 used calculated IC_50_ values using the log (test compound concentration) vs normalised response (100–0%) with variable slope formula and the least squares fitting method (i.e., *y* = 100/(1 + 10^((LogIC_50_ − *x*) × HillSlope)).

### 4.7. Research Ethics

All five medicinal plants studied here were collected from Bhutan in 2009 and in a study approved by Menjong Sorig Phamaceuticals of Bhutan and the National Biodiversity Centre of Bhutan (NBCB). A Material Transfer Agreement was signed between Phurpa Wangchuk and the NBCB and the methanol extract samples of these plants were transferred to Australia with approval from Bhutan Agriculture and Food Regulatory Authority and Australian Quarantine Authority. Their voucher specimen number and parts collected are given in Table 1. The DC2.4 cell line was generously provided by Kenneth L. Rock (University of Massachusetts Medical School and Dana-Farber Cancer Institute, USA). The non-malignant cholangiocyte cell line H69 was obtained from Gregory J. Gores, Mayo Clinic, Rochester, MN, USA.

## 5. Conclusions

Five medicinal plants—*A. laciniatum*, *A.* *nubigena*, *C. crispa*, *C. dubia*, and *P. amabile*—which grow in extreme Himalayan mountain ecology—are used in the scholarly Bhutanese traditional medicines for treating various disorders that bore relevance to modern disease pathologies including inflammation, tumor and infections. Inspired by the strong bioactivities of their crude extracts, we isolated a total of 40 phytochemicals and tested 14 of them for their capacity to modulate DC activity, and 10 for cytotoxicity. Two of the 14 compounds showed robust immunostimulatory or immunosuppressant activities. Specifically, scoulerine (**10**) showed strong immunostimulatory activity as indicated by upregulation of gene and protein expression of key molecules associated with DC activity, whereas bergapten (**13**) consistently suppressed expression of DC genes involved in T cell signalling and activation. Our data are consistent with reports that these two compounds have broad-ranging therapeutic properties that can be used against cancer, malaria, helminthiasis, inflammation and Alzheimer’s disease. Thus, they are worthy of further exploration either as independent drug entities or as immunomodulators to potentiate other drugs/vaccines, including investigating the mechanisms of action of these compounds. Finding a broad-spectrum drug that could treat multiple diseases or potentiate other drugs and vaccines is highly desirable and of great interest to the pharmaceutical industry. This study not only identified potential immunomodulatory compounds from five Bhutanese medicinal plants but also provided molecular and immunological data to support their reported efficacy.

## Figures and Tables

**Table 1:** Five medicinal plants and the selected compounds screened for immunomodulatory bioactivity in DC2.4 murine dendritic cell line and cytotoxicity in immortalized non-cancerous H69 human cholangiocyte cell line.

Botanical Name	Voucher Specimen Number	Traditional Uses [36]	Parts Used	Total Compounds Isolated and the Weight Obtained [31,32,33,34,35]	Class of Phytochemical	Major Compounds Selected for DC and Cytotoxicity Assays
*Aconitum laciniatum* (Ranunculaceae)	93	Parasite infections, leprosy, bone diseases, mumps and gout	Tuber	Pseudaconitine (1.9 g), 14-veratroylpseudaconine (28.7 mg), 14-*O*-acetylneoline (46.9 mg), neoline (428.5 mg), senbusine A (13.8 mg)	Diterpenoid alkaloids	Pseudaconitine (**1**) 14-veratroylpseudaconine (**2**) 14-*O*-acetylneoline (**3**)
*Ajania nubigena* (Asteraceae)	73	Allays abscess, swelling, tumor, fever, coughs, epistaxis and kidney infection	Aerial	Linalool oxide acetate (1.2 g), chamazulene (2.6 mg), (*E*)-spiroether (87.0 mg), (*Z*)-spiroether (6.7 mg), *p*-hydroxyacetophenone (11.3 mg), oxyanin B (18.6 mg), luteolin (618.0 mg), luteolin-7-*O*-β-d-glucopyranoside (41.3 mg)	Terpenes and flavonoids	Linalool oxide acetate (**4**) (*E*)-spiroether (**5**) luteolin (**6**) luteolin-7-*O*-β-d-glucopyranoside (**7**)
*Corydalis crispa* (Fumariaceae)	78	Allays blood, liver and bile disorders, and febrifuge	Whole	Protopine (1 g), 13-oxoprotopine (17.6 mg), 13-oxocryptopine (4.5 mg), stylopine (5 mg), coreximine (1 mg), rheagenine (1 mg), ochrobirine (60.6 mg), sibiricine (0.8 mg), bicuculline (8 mg)	Isoquinoline alkaloids	Protopine (**8**) ochrobirine (**9**)
*Corydalis dubia* (Fumariaceae)	14	Allays neuralgia, tuberculosis, and blood, liver, heart, lung, pancreas and kidney infections	Whole	Dubiamine (6.9 mg), scoulerine (9.4 mg), cheilanthifoline (15.1 mg), protopine (160 g), capnoidine (80.3 mg), bicuculline (18.3 mg), corydecumbine (12.3 mg), hydrastine (1.3 mg)	Isoquinoline alkaloids	Scoulerine (**10**) capnoidine (**11**)
*Pleurospermum amabile* (Umbelliferae)	29	Anti-dote, febrifuge, and dyspepsia	Aerial	(*E*)-isomyristicin (185.3 mg), (*E*)-isoapiol (30.7 mg), methyl eugenol (44.7 mg), (*E*)-isoelemicin (3.4 mg), psoralen (23.8 mg), bergapten (2.5 g), isoimperatorin (143.1 mg), isopimpinellin (93.8 mg), oxypeucedanin hydrate (109.7 mg), oxypeucedanin methanolate (295.2 mg)	Phenylpropanoids and furanocoumarins	(*E*)-isomyristicin (**12**) bergapten (**13**) isoimperatorin (**14**)

**Table 2:** Immunomodulatory bioactivity of 14 plant compounds in DC2.4 dendritic cell line (FACS, PCR, SUM and Average).

Comp.	Conc. (µg/mL)	% Live Cell (FACS)	FACS								PCR								SUM FACS + PCR	Ave.
			MHC-I	MHC-II	CD40	CD44	CD80	CD86	CD274	SUM	MHC-I	MHC-II	CD40	CD44	CD80	CD86	CD274	SUM		
**1**	316.00	99.20	0.90	1.10	1.17	1.09	1.33	0.57	1.22	7.38	0.83	0.57	1.32	1.56	1.86	1.37	1.29	8.80	16.18	1.16
**2**	1000.00	94.20	0.88	2.60	3.15	0.92	2.64	0.59	3.56	14.34	1.08	0.39	1.04	0.58	1.79	1.87	1.61	8.37	22.71	1.62
**3**	1000.00	94.20	0.69	2.75	3.68	1.05	3.38	0.96	5.56	18.07	0.81	0.50	2.94	0.69	1.19	2.87	3.01	12.01	30.07	2.15
**4**	316.00	97.20	0.90	1.29	2.10	1.29	1.71	1.33	1.78	10.39	1.35	0.28	1.52	1.57	2.91	2.36	2.64	12.63	23.02	1.64
**5**	100.00	94.10	1.08	3.42	1.54	1.78	3.29	1.37	3.24	15.72	0.81	0.45	0.76	1.15	1.48	1.78	1.13	7.57	23.28	1.66
**6**	10.00	95.70	0.50	1.92	1.11	1.17	1.66	1.25	1.47	9.08	1.05	1.36	0.57	1.30	1.60	1.07	1.82	8.78	17.86	1.28
**7**	100.00	96.50	0.78	1.64	1.19	1.42	1.24	1.35	1.17	8.80	1.05	0.41	0.71	1.18	1.67	1.00	3.28	9.29	18.09	1.29
**8**	100.00	93.60	0.94	1.31	1.38	1.53	1.95	1.17	1.67	9.95	0.84	3.35	1.11	0.73	1.64	1.29	1.07	10.01	19.96	1.43
**9**	100.00	97.90	0.87	1.55	1.16	1.08	1.19	0.91	1.67	8.43	0.79	1.52	1.36	1.45	2.03	1.61	2.64	11.41	19.84	1.42
**10**	31.60	83.80	2.07	2.03	2.12	2.15	4.89	2.48	3.91	19.64	1.43	1.17	1.40	3.47	5.10	2.19	2.71	17.48	37.12	2.65
**11**	316.00	96.80	1.08	1.23	2.43	1.78	1.71	1.47	2.99	12.70	0.83	2.66	1.65	1.66	2.09	1.02	2.99	12.89	25.59	1.83
**12**	100.00	92.00	1.53	1.29	2.90	1.84	1.91	2.46	2.07	14.00	1.05	0.72	1.40	1.43	2.09	1.56	1.03	9.28	23.27	1.66
**13**	100.00	86.90	0.41	2.08	1.72	1.38	1.12	1.24	2.76	10.70	0.77	0.78	1.30	0.73	0.90	1.08	2.17	7.72	18.41	1.32
**14**	31.60	95.40	0.61	1.05	1.52	1.51	1.42	1.12	1.44	8.68	0.94	0.50	1.15	1.67	1.83	1.15	2.90	10.13	18.81	1.34
**CT**	1.00	92.70	2.56	2.32	0.88	1.15	8.25	2.65	3.86	21.67	1.04	0.34	0.31	1.11	6.00	4.32	4.54	17.66	39.33	2.81

Abbreviations: Comp. = Compounds, Conc. = Concentration, Ave. = Average, CT = Cholera toxin.
